# Using Residential History and Groundwater Modeling to Examine Drinking Water Exposure and Breast Cancer

**DOI:** 10.1289/ehp.0901547

**Published:** 2010-02-17

**Authors:** Lisa G. Gallagher, Thomas F. Webster, Ann Aschengrau, Verónica M. Vieira

**Affiliations:** 1 Department of Environmental Health and; 2 Department of Epidemiology, Boston University School of Public Health, Boston, Massachusetts, USA

**Keywords:** breast cancer, GIS, groundwater, historical exposure, mobility, space-time

## Abstract

**Background:**

Spatial analyses of case–control data have suggested a possible link between breast cancer and groundwater plumes in upper Cape Cod, Massachusetts.

**Objective:**

We integrated residential histories, public water distribution systems, and groundwater modeling within geographic information systems (GIS) to examine the association between exposure to drinking water that has been contaminated by wastewater effluent and breast cancer.

**Methods:**

Exposure was assessed from 1947 to 1993 for 638 breast cancer cases who were diagnosed from 1983 to 1993 and 842 controls; we took into account residential mobility and drinking water source. To estimate the historical impact of effluent on drinking water wells, we modified a modular three-dimensional finite-difference groundwater model (MODFLOW) from the U.S. Geological Survey. The analyses included latency and exposure duration.

**Results:**

Wastewater effluent impacted the drinking water wells of study participants as early as 1966. For > 0–5 years of exposure (versus no exposure), associations were generally null. Adjusted odds ratios (AORs) for > 10 years of exposure were slightly increased, assuming latency periods of 0 or 10 years [AOR = 1.3; 95% confidence interval (CI), 0.9–1.9 and AOR = 1.6; 95% CI, 0.8–3.2, respectively]. Statistically significant associations were estimated for ever-exposed versus never-exposed women when a 20-year latency period was assumed (AOR = 1.9; 95% CI, 1.0–3.4). A sensitivity analysis that classified exposures assuming lower well-pumping rates showed similar results.

**Conclusion:**

We investigated the hypothesis generated by earlier spatial analyses that exposure to drinking water contaminated by wastewater effluent may be associated with breast cancer. Using a detailed exposure assessment, we found an association with breast cancer that increased with longer latency and greater exposure duration.

Prior spatial analyses of two population-based case–control studies of breast cancer in upper Cape Cod, Massachusetts (Figure 1) used generalized additive models and geographic information systems (GIS) to map statistically significant areas of increased and decreased odds ratios (ORs) (Vieira et al. 2005, 2008). Associations between geographic location and breast cancer diagnosed from 1983 to 1993 were highest when 20 years of latency was assumed. To generate potential hypotheses for further investigation, areas of increased ORs were compared with environmental data available from the Massachusetts GIS portal (http://www.mass.gov/mgis). A geographic overlap was observed between breast cancer and groundwater plumes contaminated by landfills and wastewater. The boundaries of these groundwater plumes were based primarily on knowledge of area groundwater flow and were developed as a worst-case scenario for contamination around each site (Eichner E, Cape Cod Commission, personal communication).

Based on the locations of public and private wells, we determined that the Barnstable Water Pollution Control Facility (BWPCF) was the only source of wastewater effluent with the potential to impact the drinking water of this study population. There are three public drinking water wells for the Barnstable Water Company (BWC) and several private wells located within the suspected plume boundary (Figure 2). Wastewater is discharged from BWPCF to the groundwater, which flows generally southeasterly toward the ocean. The existence of a sewage plume has been documented by town sources and other research (Cambareri 1986; SEA Consultants 1985; Town of Barnstable 1983a, 1983b). Groundwater wells in upper Cape Cod draw drinking water from a highly permeable aquifer that is vulnerable to contamination (Janik 1987; Zoto and Gallagher 1988). Concern for the vulnerability of this aquifer has led to recent surveys of local wastewater and groundwater, where suspected endocrine disruptors, including alkylphenols and other estrogenic phenolic compounds, have been detected (Rudel et al. 1998; Zimmerman 2005). Although there is no information on what contaminants may have been in the plumes historically, the low pH and organic carbon content of the Cape Cod aquifer enables many sewage contaminants to persist for up to 30 years in the aquifer (Barber et al. 1988).

The geographic relationship between breast cancer clusters and groundwater plumes alone does not establish exposure. The objective of this paper was to use an extensive exposure model to test the hypothesis that drinking water contaminated by municipal wastewater effluent from the BWPCF may be associated with breast cancer. We used residential histories from 1943 to 1993, questionnaire data on self-reported sources of drinking water (public or private) and on the use of bottled water, operating information for the BWC and BWPCF, and a modified three-dimensional groundwater model from the U.S. Geological Survey (USGS) to estimate when exposure occurred and which participants were exposed. Because the risk of breast cancer may be dependent on the timing of exposure in relation to diagnosis, we examined varying latency periods and exposure durations.

## Materials and Methods

### Study population

We investigated the association between exposure to drinking water contaminated by effluent from the BWPCF and breast cancer in upper Cape Cod, Massachusetts, using data from two population-based case–control studies: the Upper Cape Cancer Study (Aschengrau et al. 1998) and Women’s Health on Cape Cod Study (Aschengrau et al. 2003). The two studies had a combined total of 638 women who had been diagnosed with breast cancer and 842 controls who had been permanent residents of five towns in upper Cape Cod, Massachusetts. We used the Massachusetts Cancer Registry to identify incident breast cancer cases diagnosed between 1983 and 1993. Demographically similar women who also lived in upper Cape Cod were selected as controls by random digit dialing; Medicare records; and records of deceased residents provided by the Massachusetts Bureau of Health, Statistics, Research, and Evaluation. Cases and controls were comparable in age and vital status. We randomly assigned index years to controls in a distribution similar to that of diagnosis years for cases and used the diagnosis and index years to define the relevant time and duration of environmental exposure among cases and controls, respectively. Study participants provided informed consent, and extensive interviews by trained personnel collected information on demographic characteristics and breast cancer risk factors, sources of drinking water (public or private), and residential histories over a 40-year period. Next of kin served as proxies for cases or controls who were deceased or too ill to participate in the interview. All residential addresses in upper Cape Cod were geocoded using GIS (ESRI, Redlands, CA). A detailed account of the methods has been reported previously (Aschengrau et al. 1998, 2003). The Institutional Review Board of Boston University Medical Center approved this research.

### Exposure assessment

**Figure 2:**
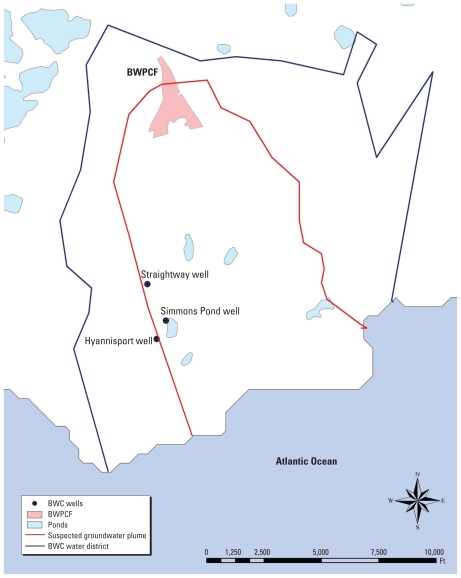
BWPCF and public drinking water wells. The map shows the locations of three BWC public drinking water wells in relation to the wastewater facility, suspected groundwater plume, and approximate water district boundary. Private drinking water wells are not shown to maintain confidentiality.

Exposure was assessed using residential histories, questionnaire data, information on the public water supply, and a three-dimensional groundwater model to track the movement of effluent from the BWPCF to the private and public drinking water wells of the study population over time. The BWPCF, operating since 1937, used primary treatment and sand filter beds until around 1980, when secondary treatment was added, before discharging effluent to the groundwater. Approximately 1.5 miles (2.4 km) south of the BWPCF are three public drinking water wells (Hyannisport, Simmons Pond, and Straightway) for the BWC, the public water supplier for hundreds of area residences (Figure 2). The BWC water district has operated since the 1920s, growing from 2 to 12 public drinking water wells between 1920 and 2005; water drawn from all the wells is mixed in common storage tanks (standpipes). Pumping reports from the 1970s and 1980s estimated that these three wells contributed 40–50% of the water supplied by the water company (Bratton 1991; LeBlanc et al. 1986).

The first aim of the exposure assessment was to determine when the drinking water wells were impacted by effluent from the BWPCF. Concerns with the vulnerability of the drinking water prompted the USGS, in cooperation with state and local agencies, to create a detailed groundwater model of the aquifer in upper Cape Cod, an area called the Sagamore Lens (Walter and Whealan 2005). This three-dimensional, finite-difference numerical model solves groundwater flow equations by representing a hydrologic system as a series of discrete spatial units within MODFLOW, a modular software program publicly available from the USGS Web site (USGS 2008). The model accounts for both horizontal movement of the plume, as well as plume depth in relation to locations of drinking water wells. Uniform geological properties and stresses, such as well pumps, are assigned to each cell of the model grid to simulate steady-state or transient flow conditions (USGS 2008; Walter and Whealan 2005). We obtained instructions from the USGS to download the necessary MODFLOW input files from the regional office (Walter D, USGS, personal communication). We used MODFLOW-GUI v4 (USGS, Reston, VA), a graphical user interface, with Argus ONE (Argus Holdings, Herzelia, Israel) to facilitate our analysis and display results with GIS (Winston 2000). Most USGS files were directly imported to this interface, but some files were recreated manually because of software limitations.

The USGS model was created for conditions in 2003, so modifications were necessary to examine historical groundwater movement. We collected data on the history of the public water system from water department personnel, including estimated start dates for wells near the facility. The Hyannisport and Simmons Pond wells came online in 1953 and 1970, respectively, and the Straightway well came online in 1961 and was replaced in 1984 (Condrey D, personal communication). Along with the start of facility operations in 1937 and the last year (1993) in which women with breast cancer were identified, these dates define five distinct time periods (1937–1953–1953–1961–1961–1970–1970–1984–1984–1993) with varying well pumping conditions for modeling groundwater flow.

Limited historical data included reports of total withdrawals for all three wells together and pumping rates after the shutdown of the 1961 Straightway well (Barlow 1995; Bratton 1991; LeBlanc et al. 1986). Pumping rates for 2003 that were used in the USGS model for the Hyannisport and Simmons Pond wells were retained for the historical model. These values seemed reasonable for normal operations over the long period, at 30% and 50% of each pump’s maximum rated capacity, respectively, and were plausible given the total withdrawals in the mid-1970s (SEA Consultants 1985). The depth and pumping rate for the 1961 Straightway well was unknown, because it was not part of the 2003 USGS model (the 2003 USGS model included the 1984 replacement Straightway well). We included the 1961 Straightway well in our model at the same depth as nearby wells, a likely position indicated by earlier studies (SEA Consultants 1985). Groundwater flow is highly dependent on pumping rates, so as a sensitivity analysis, three scenarios were modeled for the Straightway well with varying percentages of the rated capacity for the well (SEA Consultants 1985): 30% capacity (28,875 ft^3^/day), 50% capacity (48,125 ft^3^/day), and 75% capacity (72,188 ft^3^/day).

Once groundwater flow was calculated in MODFLOW, the results were used by the MODPATH program (version 4.3; USGS) to delineate the path of effluent particles by advection over time to the public and private drinking water wells (Winston 2000). Using the same methodology as earlier USGS studies, particle movement in groundwater from the BWPCF was tracked beginning in 1937 to determine if and when those particles reached the wells for each pumping scenario (Barlow 1995; Walter and Whealan 2005). Groundwater containing effluent was determined to have reached a well when a particle track ended in the model grid cell containing the well. The exposure assessment assumed effluent moved at the same rate as the groundwater. The study participants served by the BWC were assumed to be equally exposed, because the distribution system stores and draws mixed water from a common standpipe (Condrey D, personal communication).

Questionnaire data were used to determine the drinking water source (public or private) of the participants for each of their residences. For participants who reported public drinking water, GIS files of the BWC pipe distribution system and geocoded addresses were used to identify which participants were supplied by BWC. Private drinking water wells were assumed to be impacted by the plume if a particle was tracked within a depth of 100 ft (30.5 m) of the residence of the subject. This criterion was used because wells in this type of aquifer in Massachusetts are commonly < 100 ft deep (Tomlinson 2008). Participants were also asked if they regularly used bottled water, although this information was not provided by residence. Consequently, bottled water use was examined by stratifying the analysis on whether women ever regularly used bottled water.

Lastly, residential histories were used to identify which participants were living at residences during years when drinking water was impacted by effluent. We defined ever exposed as one or more years at a residence supplied by BWC or by an impacted private well. Cumulative duration of exposure was calculated by adding the number of ever-exposed residence years for all addresses.

### Data analysis

The epidemiological analysis first compared ever-exposed women with never-exposed women. Never-exposed women obtained their drinking water from public or private wells in upper Cape Cod that were not impacted by effluent from the BWPCF. We also compared women by duration (years) of exposure to an affected private or public well and stratified by regular bottled water use. Multivariable logistic regression was used to estimate ORs and control for covariates. The OR was estimated using the antilog of the beta coefficient. Statistical stability was evaluated with a 95% confidence interval (CI). Adjusted analyses controlled for age at diagnosis or index year (< 49, 50–59, 60–69, 70–79, ≥ 80), vital status at interview, family history of breast cancer based on first-degree relatives, personal history of breast cancer (before current diagnosis or index year), age at first live birth or stillbirth (< 30 years, ≥ 30 years, nulliparous), education level (< 12 years, 12 years, or > 12 years), race (white or other), and study population (i.e., study 1 or 2). Additionally, we adjusted for ever/never exposure to tetrachloroethylene (PCE) from leaching vinyl-lined drinking water pipes, another contaminant in the public water supply for both study populations, and duration of residence in Cape Cod. Positive associations with breast cancer were previously observed for women exposed to PCE-contaminated drinking water (Aschengrau et al. 2003) and women with longer durations of residence in Cape Cod (McKelvey et al. 2004). The leaching and transport algorithm used to estimate exposure to PCE in drinking water was based on residential history and characteristics of the water distribution system and were described in earlier studies (Aschengrau et al. 2008, 2009; Janulewicz et al. 2008). We also considered a range of latent periods (i.e., 0, 10, 15, and 20 years), because it was possible that the effluent contained pollutants, such alkylphenols and other estrogenic phenolic compounds, that may either initiate or promote breast cancer.

## Results

Participants were predominantly white, > 60 years of age, and had > 12 years of education. Most were alive at interview; 30% of cases and 40% of controls were deceased and their information was collected by proxy. More cases than controls had first-degree family history of breast cancer, were ≥ 30 years old at first birth, or were nulliparous (Aschengrau et al. 1998, 2003).

**Figure 3:**
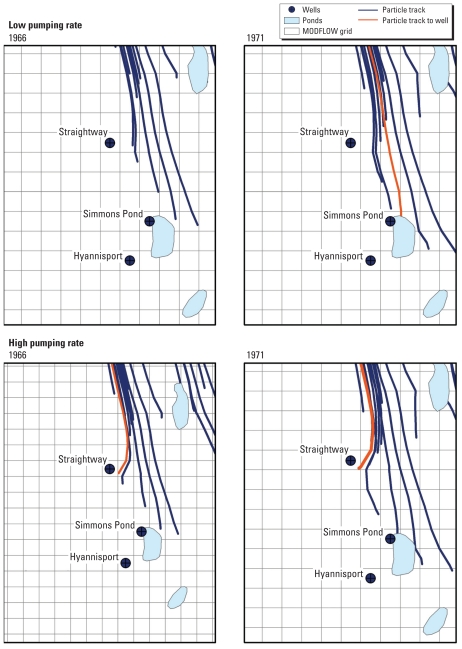
Particle-tracking analysis for groundwater originating at BWPCF. Groundwater containing effluent was determined to have reached the well when a particle track ended in the model grid cell containing the well. If the 1961 Straightway well was pumping at a low rate, groundwater from the BWPCF from start of its operation in 1937 would have reached the Simmons Pond public drinking water well in 1971. At a higher pumping rate, contaminated groundwater would have reached Straightway well in 1966. These 2 years define the start of exposure in analyses for the low and high pumping rates.

Groundwater modeling indicated that effluent from the BWPCF reached drinking water wells for the public water supplier BWC as early as 1966. The sensitivity analysis to estimate groundwater flow indicated that effluent would have arrived at different times depending on the pumping rate for the original Straightway well (Figure 3). Assuming a high pumping rate of 75% capacity (72,188 ft^3^/day), effluent would have reached the original Straightway well in 1966 and the Simmons Pond well in 1973. Assuming a low pumping rate of 30% capacity (28,875 ft^3^/day), the Simmons Pond well would have been impacted first, and not until 1971, and the replacement Straightway well would have been reached around 1989. The results for 50% capacity were similar to those for 30% capacity (data not shown).

Because exposure would have begun in 1971 with the low pumping rate and in 1966 with the high pumping rate, there was a 5-year difference in opportunity for exposure. The low and high pumping scenario predicted similar numbers of ever-exposed subjects (*n* = 241 and *n* = 247, respectively) when latency was ignored (Table 1). However, when latency was considered, fewer subjects were classified as exposed when the lower pumping rate was assumed (Table 1). This finding was mainly because the case ascertainment period was 1983–1993, so when we considered a 20-year latency period, only those participants with a diagnosis or index year during 1991–1993 could have been exposed in the low pumping rate scenario, whereas participants with a diagnosis or index year from 1986 to 1993 may have been exposed in the high pumping rate scenario.

Without considering latency and irrespective of the pumping rate, approximately 78–80% of the subjects were exposed at one address, 17–19% were exposed at two to three addresses, and 3% were exposed at four to six addresses. The overall distribution of exposure duration was similar with each pumping scenario, but the distribution varied over latency periods (Table 1). Subjects had a cumulative exposure from 1 year to approximately 20–40 years, depending on latency period. Based on the distribution of exposure durations, associations with breast cancer were estimated for > 0 to 5 years, > 5 years, and > 10 years of exposure relative to no exposure at any time during the study period.

When latency was not considered, there were small increases in breast cancer ORs for participants exposed > 10 years compared with subjects who were never exposed [adjusted odds ratio (AOR) = 1.3; 95% CI, 0.9–2.0 for low pumping rate and AOR = 1.3; 95% CI, 0.9–1.9 for high pumping rate] (Table 2). When a 10-year latency period was considered, larger ORs were seen for the subjects exposed > 5 years (AOR = 1.5; 95% CI, 0.9–2.7 and AOR = 1.4; 95% CI, 0.9–2.2 for low and high pumping rates, respectively). The same was true for exposure > 10 years (AOR = 1.6; 95% CI, 0.8–3.2 for high pumping rate), although AORs could not be calculated for the low pumping rate scenario because of small numbers [crude odds ratio (COR) = 4.6; 95% CI, 1.0–22.3]. Small numbers also limited the adjusted analysis when 15- and 20-year latency periods were considered, but positive associations were seen for ever-exposed versus never-exposed subjects in either pumping scenario (CORs = 1.2–5.9).

A total of 342 participants reported ever regularly drinking bottled water, and 1,138 reported not doing so. Across all latency periods and pumping rates, we found statistically significant associations for ever-exposed versus never-exposed participants among women who did not regularly use bottled water and nonstatistically significant inverse associations among women who did regularly use bottled water. The strongest association was observed for women who did not use bottled water when 20 years’ latency and a high pumping rate were assumed (AOR = 2.5; 95% CI, 1.2–5.3 and AOR = 1.0; 95% CI, 0.3–3.3 for nonbottled water users and bottled water users, respectively).

## Discussion

In this study, we developed a historical groundwater model and examined participants’ residential histories and drinking water source to determine exposure to drinking water contaminated by effluent from the BWPCF. Recent space–time analyses showed increased breast cancer ORs among women who lived near the BWPCF compared with women who lived in other parts of the study area during the 1960s (Vieira et al. 2008). We used an existing groundwater model and particle-tracking analysis to estimate the movement of effluent discharge from the BWPC facility to the drinking water wells. Although several assumptions were required to address historical conditions, the model provided a method to explore the spatial and temporal relationship between a source of contamination and a possible exposure route for study participants. We found increased breast cancer for subjects ever exposed to contaminated drinking water wells when considering various latency periods, but little to no evidence of an association when latency was ignored.

The BWPCF was and continues to be a major source of groundwater recharge in upper Cape Cod. Evidence of this is provided by drinking water quality measurements of nitrate-nitrogen (nitrate-N) collected since the Safe Drinking Water Act was promulgated in 1972 [Janik 1987; Rask and Bourne 1998; SEA Consultants 1985; Silent Spring Institute (SSI) 2008; Swartz et al. 2003)]. Nitrate-N is an indicator of contamination from septic systems or wastewater recharge that may include potentially harmful contaminants such as synthetic organic chemicals (Janik 1987). Nitrate-N concentrations in drinking water wells in Cape Cod < 0.5 mg/L are considered background levels, and concentrations > 1.0 mg/L are due to human impact, typically from wastewater or fertilizer use. The U.S. Environmental Protection Agency maximum contaminant level for nitrate-N is currently 10 mg/L (Rask and Bourne 1998). With the exception of a few years, the nitrate-N levels at the public drinking water wells near the BWPCF were > 1.0 mg/L and often approached 5 mg/L from the early 1970s to 1990s (Janik 1987; SSI 2008). The overall trend in the monitoring data shows that nitrate-N measurements increased at a similar rate as the quantity of discharged effluent over time. This suggests that effluent impacted the monitoring wells, although increases in residential septic use may also have contributed to these increases (Barlow 1995). Because historic monitoring was only of nitrate-N, we do not know what chemicals may have been present in the effluent when contamination of the drinking water wells first occurred in the late 1960s and early 1970s.

Results from our groundwater model were comparable to earlier studies with regard to plume distance and depth, but differed in direction. In the mid-1980s, studies documented that the sewage plume extended 5,500 ft (1,646.4 m) south of the facility, with a depth of 20–65 ft (7.6–19.8 m) below the surface bounded vertically by an impermeable clay deposit (Cambareri 1986; SEA Consultants 1985; Town of Barnstable 1983a, 1983b). The depth of the plume was within the range of the BWC wells [between 61 and 75 ft (18.6– 22.9 m) deep], although there was some evidence that the deepest well, the Simmons Pond well, may have penetrated a lower aquifer system and thus was protected from contamination above by the layer of clay (SEA Consultants 1985). However, the earlier studies concluded that several ponds in the area directed the wastewater plume toward the harbor, completely by-passing the public drinking water wells (Town of Barnstable 1983a, 1983b). The surface water interactions that we incorporated into our historical groundwater model indicates this may have protected the Hyannisport well, but the Straightway and Simmons Pond wells may still have been affected (Figure 3). Our model found an overall larger area impacted by groundwater originating at the facility than was delineated by these earlier plume studies and thus included the drinking water wells. The model also found it took approximately 30 years for the effluent to reach the public drinking water wells. A sewage plume in the nearby town of Falmouth showed similar persistence and behavior under similar geologic conditions (LeBlanc 1984).

In the 1980s, low levels of other wastewater indicators (specific conductance, ammonium, alkalinity, and potassium) at monitoring wells 2,000 ft (609.6 m) north of the drinking water wells were cited as evidence that the plume was not moving in the direction of public drinking water wells but was passing southeasterly to the ocean (Cambareri 1986). However, some uncertainty surrounds this conclusion because these samples did not represent historical conditions or conditions at the drinking water wells located to the south. At the time of the studies in the 1980s, additional monitoring was recommended to establish the source of the elevated nitrate-N measurements, as the existing monitoring wells were considered to be insufficient (Barlow 1995; Cambareri 1986; SEA Consultants 1985). It is possible that monitoring in earlier studies did not adequately delineate the edges of the Barnstable plume and its southerly extent, so drinking water wells may have been impacted by the sewage plume in prior decades as our model results suggest (Cambareri 1986; Town of Barnstable 1983a, 1983b).

To the best of our knowledge, only one other epidemiologic study has investigated the association between breast cancer and exposure to drinking water potentially contaminated by wastewater (Brody et al. 2006; Swartz et al. 2003). That study, which was also conducted among women living in Cape Cod, used nitrate-N levels measured in public water supply wells beginning in 1972 as a measure of exposure to public drinking water contaminated by onsite wastewater disposal (septic systems). The study did not find evidence of an association with breast cancer after considering a range of latent periods. Differences in study methods, including exposure definitions, may account for the dissimilar findings. In the Brody et al. study (2006), cancer cases were diagnosed between 1988 and 1995 while living in one of the 15 Cape Cod towns. Because the exposure data only went back to 1972, participants were included in the study only if they had lived in Cape Cod on a public drinking water supply during the 16-year period prior to diagnosis (or index year for controls) (Brody et al. 2006). In contrast, our study examined a smaller region of Cape Cod using cases diagnosed between 1983 and 1993. We also included both public and private well users and modeled exposure beginning in 1966. The main difference is that the Brody et al. study assessed the widespread exposure to public drinking water impacted by residential septic systems, whereas the current analysis assessed exposure to effluent from the BWPCF. The Barnstable treatment facility is a considerably larger point source, concentrating waste from across a broad geographic area and combining both residential and commercial wastewater.

This study investigated a hypothesis generated by prior spatial analyses. An important strength of this analysis was the use of various data sources to reconstruct historical exposures with respect to both space and time. We integrated groundwater modeling, residential mobility, and information about public water systems in GIS to assess exposure to drinking water impacted by wastewater effluent. Despite our best efforts in modeling groundwater movement and determining who was exposed, the analysis is still limited by exposure misclassification. Assumptions concerning operations of the public water system, wastewater facility, and groundwater conditions were necessary. Our sensitivity analysis showed that the exposure period depended on pumping rate conditions for a public well with missing information. In addition, unknown changes over time in drinking water withdrawals, aquifer conditions (i.e., precipitation, impact of facility effluent discharge on groundwater levels), distribution system infrastructure (adding pipes, water storage, mixing), and personal water usage also affected the exposure of subjects at their residences. The misclassification of exposure status, however, was likely nondifferential, as both cases and controls in this area were served by the same supply. Another limitation is that the composition of the wastewater effluent was unknown, other than some compounds such as nitrate-N after 1972. Thus, we could not account for reactions within the plume and aquifer that may have slowed the movement of contaminants through the aquifer. In addition, we could not account for changing concentrations over time. However, we conducted an additional analysis using nitrate-N concentrations at the drinking water wells as an indicator of trends in the concentrations of other potential contaminants. We created a scaled relative measure of exposure by adding available nitrate-N concentrations at the three Barnstable wells over the residence period for exposed subjects (SSI 2008). For a 10-year latency with low pumping rate scenario, the AOR was 1.7 (95% CI, 0.9–3.4) for the highest nitrate-N exposure quartile (versus no exposure), which is similar to the association with > 5 years of exposure (versus no exposure) to the contaminated plume (AOR = 1.5; 95% CI, 0.9–2.7). We found the same association with this nitrate-N measure across latency periods. We feel these results support our original assumption that exposure is driven by duration relative to the timing of effluent reaching the well.

The current study results may also have been affected by uncontrolled confounding by other environmental exposures. For example, the first Straightway well was closed because of contamination by volatile organic compounds, with little further explanation (Town of Barnstable 1997). In addition, the 1982 Upper Cape Cancer Incidence Study, which examined cancer in relation to many different environmental exposures, found it difficult to separate exposure to the BWC and the Barnstable Airport (Aschengrau and Ozonoff 1982). Most individuals served by the BWC also live in proximity to the airport. That study did not find an association between breast cancer and contaminated drinking water from BWC, but the case ascertainment period was 1983–1986 and few participants were exposed for long durations. There may also have been uncontrolled confounding from pesticide exposure from cranberry bog cultivation. We did address PCE contamination and residency duration as confounders in our analysis, but they did not affect the results and were not included in the final model. We also estimated associations with breast cancer after excluding the 86 women with a prior history of breast cancer and observed similar point estimates and CIs (data not shown). It is unlikely that the results were biased by missing data, as residential mobility and water type were reported with similar completeness for cases and controls. In addition, associations with established breast cancer risk factors were present in the study populations, as previously described (Aschengrau et al. 2008, 2009; Janulewicz et al. 2008).

When cancer clusters are discovered, there are many possible environmental factors that could be investigated. In this study, given the geographic overlap of groundwater plumes identified using spatial analyses, we investigated the hypothesis that exposure to drinking water contaminated by effluent from a wastewater treatment facility was associated with breast cancer. The spatial relationship alone does not establish exposure, but the current study was able to determine a plausible route of exposure by also taking time into account. Assessment of historical environmental exposures often requires substantial effort, and the general methodology described here can be applied elsewhere. Although definitive associations were not found in this study, we showed that by incorporating additional data, such as residential histories of the participants and contaminant movement over time, hypotheses generated by spatial analyses can provide additional insights into the environmental etiology of breast cancer.

## Conclusions

This analysis found evidence of a positive association between breast cancer and exposure to drinking water impacted by wastewater effluent from the BWPCF. The associations were strongest among women who were not regular bottled water users and among women exposed for long durations when latency periods were taken into account. The current exposure analysis expands on our earlier work to explore the spatial and temporal relationship between a source of environmental contamination and a route of exposure for this study population. Our prior spatial analysis identified groundwater plumes as a potential environmental exposure that had not yet been fully studied. We investigated this hypothesis using a detailed groundwater model and determined that contamination of drinking water by effluent from the BWPCF was plausible. Area groundwater sources for drinking water are subject to more protections now, and the impact of sewage on groundwater was carefully considered in recent expansion plans for the facility. However, this analysis suggests the sewage plume emanating from the facility may have had a significant historical impact on drinking water.

## Figures and Tables

**Figure 1:**
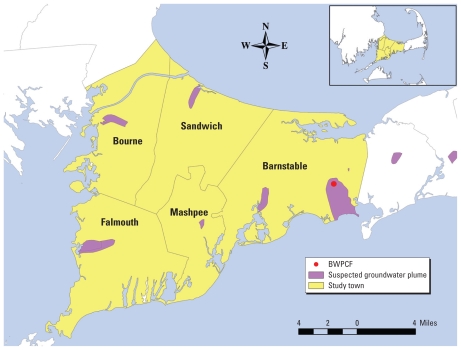
Study area in the Cape Cod region of Massachusetts. The upper region of Cape Cod consists of five towns: Barnstable, Bourne, Falmouth, Mashpee, and Sandwich. Groundwater contamination was suspected in several areas, but the BWPCF was the only source with the potential to impact drinking water in this study population.

**Table 1:** Distribution of exposure duration among exposed participants, according to latency period and pumping scenario.

Scenario	Latency (years)	Duration of exposure (years)				
		*n*	Minimum	Median	75th percentile	Maximum
Low pumping rate (exposure begins in 1971)	0	241	1	11	14	36
	10	155	1	5	6	36
	15	71	1	3	5	21
	20	11	1	2	3	17
High pumping rate (exposure begins in 1966)	0	247	1	12	19	39
	10	162	1	8	11	29
	15	122	1	5	6	24
	20	52	1	3	4	19

Max, maximum; Min, minimum.

**Table 2:** CORs, AORs, and 95% CIs for breast cancer according to duration of exposure and pumping scenario relative to participants who were never exposed.

	Low pumping rate, exposure duration				High pumping rate, exposure duration			
Latency period (years)	Ever	> 0 to 5 years	> 5 years	> 10 years	Ever	> 0 to 5 years	> 5 years	> 10 years
0								
Case/control	103/138	29/44	74/94	56/65	105/142	26/47	79/95	58/71
COR (95% CI)	1.0 (0.7–1.3)	0.9 (0.5–1.4)	1.0 (0.7–1.4)	1.1 (0.8–1.6)	1.0 (0.7–1.3)	0.7 (0.4–1.2)	1.0 (0.8–1.5)	1.1 (0.7–1.5)
AOR (95% CI)	1.2 (0.9–1.6)	1.0 (0.6–1.8)	1.2 (0.8–1.7)	1.3 (0.9–2.0)	1.1 (0.9–1.5)	0.9 (0.5–1.5)	1.3 (0.9–1.8)	1.3 (0.9–1.9)

10								
Case/control	72/83	41/56	31/27	7/2	74/88	25/32	49/56	23/19
COR (95% CI)	1.1 (0.8–1.6)	1.0 (0.6–1.5)	1.5 (0.9–2.6)	4.6 (1.0–22.3)	1.1 (0.8–1.5)	1.0 (0.6–1.8)	1.1 (0.7–1.7)	1.5 (0.9–2.9)
AOR (95% CI)	1.3 (0.9–1.9)	1.2 (0.8–1.9)	1.5 (0.9–2.7)	–	1.3 (0.9–1.8)	1.1 (0.6–1.8)	1.4 (0.9–2.2)	1.6 (0.8–3.2)

15								
Case/control	39/32	32/30	7/2	1/0	57/65	31/46	26/19	5/0
COR (95% CI)	1.6 (1.0–2.6)	1.4 (0.8–2.3)	4.6 (1.0–22.3)	–	1.2 (0.8–1.7)	0.9 (0.6–1.4)	1.8 (1.0–3.3)	–
AOR (95% CI)	1.6 (0.9–2.6)	1.4 (0.8–2.4)	–	–	1.4 (0.9–2.0)	1.1 (0.7–1.9)	1.8 (1.0–3.6)	–

20								
Case/control	9/2	8/2	1/0	1/0	31/21	25/21	6/0	1/0
COR (95% CI)	5.9 (1.3–27.5)	5.3 (1.1–24.9)	–	–	1.9 (1.1–3.4)	1.6 (0.9–2.8)	–	–
AOR (95% CI)	–	–	–	–	1.9 (1.0–3.5)	1.6 (0.9–3.1)	–	–

The low and high pumping rate scenarios assume different rates for the Straightway well. Particle-tracking analyses indicate effluent would have reached the Simmons Pond well in 1971 for the lower pumping rate and in 1966 for the higher pumping rate. Adjusted analyses controlled for age at diagnosis or index year, vital status at interview, family history of breast cancer, personal history of breast cancer (before current diagnosis or index year), age at first live birth or stillbirth, education, race, and case–control study population. Referent groups for all exposures were women who were unexposed over the entire study period. In the low pumping scenario, 704 controls and 535 cases were unexposed. In the high pumping scenario, 700 controls and 533 cases were unexposed.
